# Prevalence, impact and care of foot problems in people with rheumatoid arthritis: results from a United Kingdom based cross-sectional survey

**DOI:** 10.1186/s13047-017-0229-y

**Published:** 2017-10-27

**Authors:** Oonagh Wilson, Sarah Hewlett, James Woodburn, Jon Pollock, John Kirwan

**Affiliations:** 1 Faculty of Health and Applied Sciences, University of the West of England, Bristol, UK; 2 School of Health and Life Sciences, Glasgow Caledonian University, Glasgow, UK; 3 School of Clinical Sciences, University of Bristol, Bristol, UK

**Keywords:** Feet, Rheumatoid arthritis (RA), Prevalence, Foot care, RA population survey

## Abstract

**Background:**

Foot symptoms in rheumatoid arthritis (RA) derive from a combination of inflammation, altered foot mechanics, deformity and secondary skin lesions. Guidelines recommend regular review of patients’ feet, but the extent to which the general population of RA patients report foot symptoms and access foot care has not been established. The aims of this study were to determine the prevalence, impact and care of foot problems in all patients with RA in one geographical area and identify factors associated with accessing foot care.

**Methods:**

Cross-sectional survey of a random sample of patients with RA, who resided within a single community-based National Health Service (NHS) podiatry service. The questionnaire collected demographic data (age, gender, local deprivation score), clinical data (disease duration, arthritis medications, disability (Health Assessment Questionnaire (HAQ)), current foot problems, foot care accessed (podiatry, orthotics and/or orthopaedics) and care received, measures of impact (Foot Impact Scale) and ability to work.

**Results:**

Of 1003 total eligible patients in the target population, 739 were posted survey packs. Of these 413 (56%) replied. Responders and non-responders had similar age (63.5 yr. vs.61.5 yr), gender (74.1%F vs. 75.2%F), and highest deprivation category (13.3% vs.15.9%). Of the responders 92.1% reported current foot problems: articular 73.8%, cutaneous lesions 65.4%, structural 57.6%, extra-articular 42.6%. Responders’ median (IQR) disease duration 10 (5–20) years, HAQ 1.5 (0.75–2.0), FIS_IF_ 10 (6–14) and FIS_AP_ 16 (7–23) and 37.8% reported impacts on work. While 69.5% had accessed foot care there were differences in the route of access (by gender and whether independent or NHS provision) and were older (64.9 yr. vs 60.4 yr. *p* = 0.001), had longer disease duration (12 yr. vs 7 yr. *p* < 0.001) and had a greater proportion of females (72.2% vs 61.7% *p* = 0.04) than those who had not accessed care.

**Conclusions:**

Current foot problems were reported by 92.1% of the study sample and substantially impacted on life and work. While overall access to foot care was higher than anticipated, routes of access differed and extent of current problems suggests the provision of effective, timely and targeted care is a pressing need.

**Electronic supplementary material:**

The online version of this article (10.1186/s13047-017-0229-y) contains supplementary material, which is available to authorized users.

## Background

Foot involvement is so common in early rheumatoid arthritis (RA) that it is part of the well-described clinical picture at presentation [1]. Continuing foot involvement in patients with longstanding RA has been estimated as 30% to 90% [2–4] but no formal survey has been reported in a random selection of a geographically defined RA population. Clinical issues involving the feet include articular features such as joint pain, stiffness and swelling; extra-articular features such as bursae, nodules and numbness; structural deformities such as hallux valgus and toe deformities; and cutaneous lesions such as callosities, nail pathologies and ulceration [4–7]. Foot problems can lead to reduced walking distance, impaired health-related quality of life and an increased risk of falls [8–10]. Despite recent advances in the medical management of RA, prospective longitudinal studies report that even in patients classified as being in remission, up to 40% have continuing disease features in the feet [2, 11]. Although national guidelines and expert opinion call for timely and appropriate foot care [12–14], provision of dedicated foot care services for inflammatory arthritis is variable and service provision has been reported to be poor [15–17]. Furthermore, the non-pharmacological management of foot problems in patients with RA can involve a variety of interventions such as treatment for cutaneous lesions, provision of foot orthoses, prescribed footwear and orthopaedic surgery. These treatments can be delivered by a variety of clinicians within both primary (community) and secondary (hospital) care settings such as podiatrists, orthotists and orthopaedic surgeons. Additionally, foot care in the UK can be provided within the National Health Service (NHS) and independent health care sector (outside NHS provision). Overall access to and utilisation of foot care services is reported to be relatively low compared to foot health care needs and inequitable, being more likely to be taken up by affluent older women [18–20].

However, the evidence base quantifying the prevalence of foot problems and access to foot care in patients with RA has uncertainty, as it rests on observational hospital-based studies using convenience sampling strategies or surveys of self-selecting groups using restricted outcome measures [4, 6, 21]. Although these studies have provided valuable insights their findings cannot be extrapolated to the general RA population. Furthermore, they may not account for regional variation of foot care service provision for patients with RA [15]. In addition, the clinical features of foot problems in RA and a description of care received have not been documented in detail.

In order to establish the prevalence and impact of foot problems and access to foot care in patients with RA a survey is required of a large group of patients with RA, randomly selected from a defined population which has equitable access to both primary and secondary based foot care services and including assessment of the full range of impact. Here we report such a survey using an RA population-based sample in a well-defined geographical area to determine the prevalence of self-reported foot problems, assess their impact (based on previous qualitative work [22], identify the proportion of patients who have accessed foot care (podiatry, orthotics and/or orthopaedics) within the study geographical area.

## Methods

### Study population

The study design was an RA population-based, cross-sectional survey of patients conducted in Bristol, United Kingdom. Bristol has a mixed population with a broad range of social affluence and deprivation [23]. Rheumatology services for the city of Bristol and surrounding areas are provided by two NHS hospitals with rheumatology services and long-term follow-up of RA patients similar to those in other parts of England [24]. NHS orthotics and orthopaedic services are provided at both hospitals. A single community-based service provides NHS podiatry care to a well-defined local population based on their registration with primary care general practices all of which feed into one or both hospitals. The general practices are within the geographical boundary of the Bristol Clinical Commissioning Group (CCG) which is responsible for NHS services for the city of Bristol. The target population for this study was patients diagnosed with RA [25] attending for rheumatology medical care at either hospital, over the age of 18 years, and registered for primary care within the community service geographical boundary. Thus the target population was all adult patients with a consultant diagnosis of RA residing within the Bristol CCG geographical area. Hospital databases were accessed to facilitate identification of patients within the target population. Within the study geographical area patients are universally diagnosed with RA in specialist care (hospital based consultants). As the hospital databases cannot select on geographical area, patient details were reviewed to ensure they were within the defined community service boundary. A total of 2335 patients were registered for rheumatology medical care at the two hospital sites. Of these, 1003 RA patients (target population) were within the community service geographical boundary and therefore met the study eligibility criteria.

The study subjects were selected by random sampling from departmental databases of the target population at both hospitals. Patients were sent an invitation letter, a questionnaire and a FREEPOST return envelope. If no response was received within 3 weeks this was repeated. If any patients had a pending rheumatology appointment within the next month, they were not contacted until after that appointment. This was to reduce the potential for the invitation on accessing foot care to influence the patient’s imminent consultation, and thus alter the response to the survey on discussions about feet during consultations.

The sample size requirements for this study were difficult to estimate as the extent of variation in access to foot care (and possibly foot care needs) was unknown a priori. Based on existing published rates [4, 20, 21] the chosen target of 400 patients would enable the proportion of patients reporting foot problems or with specialist foot care need or history to be estimated with a margin of error not greater than 5 percentage points and confidence limits of at least 95%.The chosen target was also based on the expectation that there would be a maximum of 5 to 6 main determinants of access to foot care, which would be identified by multivariate analyses with adequate precision [26]. Questionnaires were distributed to RA patients in randomly assembled batches and this continued until the number of questionnaires returned reached the chosen target of 400 returned response sets. Research ethics committee approval was obtained (Central Bristol Medical Research Ethics Committee, 11/SW/0327) and informed consent was inferred by the return of completed questionnaires.

### Study questionnaire

The study questionnaire collected clinical and general demographic data, presence and impact of foot problems, foot care accessed and a description of foot care received. Responders were classified as having accessed foot care (AFC) if they reported to have accessed/utilised podiatry, orthotics and/or orthopaedics. Conversely, responders who had not accessed any of the defined foot care services were classified as not accessed foot care (NAFC). The content of the questionnaire was developed from data generated from one-to-one interviews with patients with self-reported foot problems [22], a narrative review of the literature in relation to studies reporting foot problems [27], validated questionnaires measuring the impact of foot problems in RA (Foot Impact Scale (FIS)) [28], and a measure of general disability (Health Assessment Questionnaire (HAQ)) [29, 30]. A convenience sample of 10 patients commented on the content of the study materials and provided valuable feedback to inform the final format of the questionnaire.

### Scoring the scales

The FIS comprises two subscales, FIS Impairment / Footwear (FIS_IF_) and FIS Activities / Participation (FIS_AP_). FIS_IF_ scores ≤ 6 are considered mild, 7–13 moderate and ≥ 14 severe measures of foot related impairment. FIS_AP_ scores ≤ 9 mild, 10–19 moderate and ≥ 20 severe are considered for measurements of activity limitation [5]. Full guidance on the scoring of the FIS (for example how to deal with missing data) has not been published; a pre-determined, pragmatic approach was therefore required in relation to scoring the scale. For this study a minimum of 90% of all questions had to be completed for the scores to be admissible (FIS_IF_ > 18 and FIS_AP_ > 27 questions completed). Missing values were given the average of the individual patient’s score for the other questions. If any returned FIS questionnaires were not sufficiently completed to meet the defined admissible criteria, the incomplete FIS scores were excluded from analysis. HAQ scores were admissible if there was at least one response in at least 7 sections and the missing section was given the average score of the individual patient’s score for the other sections [29].

### Social deprivation

The Index of Multiple Deprivation (IMD) 2007 for England was used as a measure of local deprivation. IMD scores were obtained from postcodes utilising GEOConvert software (http://geoconvert.mimas.ac.uk, accessed 09/10/13). IMD scores were taken for the whole study sample and were converted into categories. Category 1 of least deprived (most affluent) was defined as the lowest 20% of numerical scores recorded in the study, Category 2 represents the second fifth (21%- 40%), and up to category 5 most deprived (least affluent (81–100%). These categories are therefore an index of comparative local deprivation *within* the study sample [31].

### Statistical analysis

Descriptive statistics were used to characterise the study sample. Continuous variable data were expressed either as means, standard deviations (SD) or as medians with their inter-quartile range (IQR), depending on the underlying distribution. For categorical data, proportions were calculated and expressed as percentages.

Univariate analyses were conducted to compare the general and clinical characteristics of responders who accessed foot care (AFC) and responders who had not accessed foot care (NAFC) since being diagnosed with RA. Continuous variable data were expressed either as means, standard deviations (SD) and compared using independent sample t-tests, or as medians with their inter-quartile range (IQR) and compared using the Mann-Whitney U test, depending on the underlying distribution. For categorical data, proportions were calculated and expressed as percentages and, where appropriate, compared using the Chi-squared test applied to the original numbers. *P* values < 0.05 were considered statistically significant.

Multivariate analyses were undertaken to determine the statistical significance of contributory factors as independent variables influencing access to foot care (AFC/NAFC) as the dependent variable. Binary logistic regression was selected as the method for multivariate analyses. Initial selection of the independent variables included in the logistic regression model was conducted after univariate analyses identifying differences (non-foot related) between the AFC group and the NAFC group (NAFC). Logistic regression analyses were then performed to assess the predictive ability of each independent variable by controlling for the effects of the other independent variables in the model. Analyses were conducted utilising binary entry (block entry) whereby all the independent variables were entered in to the model simultaneously. Incomplete data sets (missing data) were excluded from logistic regression analyses using case pairwise deletion. Predictor variables associated with the outcome with *p* ≤ 0.05 were selected as variables in a series of logistic regression models. The statistical analyses were conducted using SPSS 19.0 (SPSS Inc. Chicago, Illinois).

## Results

### Participants

**Fig. 1:**
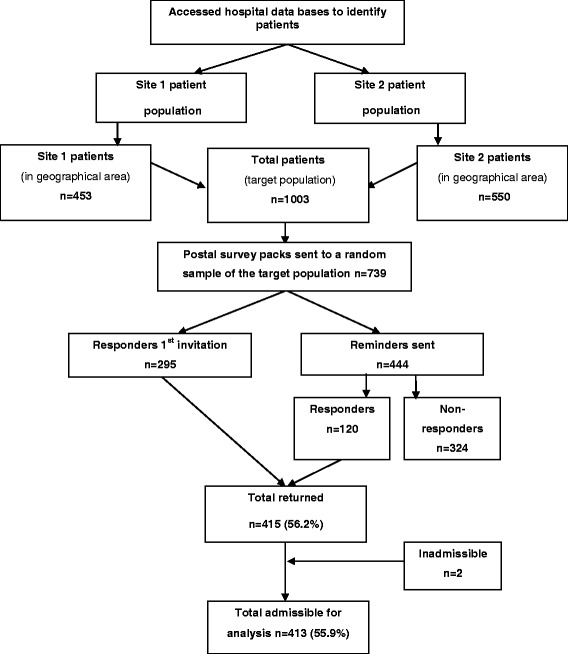
Flow diagram of study recruitment

Of the 1003 patients, within the target population, 739 were posted a questionnaire before the target sample size was achieved and 415 returned. Of these 295 were returned directly and 120 after a reminder, giving an overall response rate of 56.2% (Fig. 1). Two returned surveys were inadmissible due to large quantities of missing data and 413 were available for analysis.

Some information was available on the 324 non-responder study subjects in relation to gender, age, local deprivation scores and hospital site. The characteristics of the responders and non-responders to the questionnaire are summarised in Table 1. Responders were slightly younger than non-responders (63.5 years v 61.5 years) but a similar proportion were male (25.9% responders v 25.3% non-responders). There was only a small difference in overall local deprivation scores between responders and non-responders. Additionally response rates were very similar for both hospital sites. Responders were consequently regarded as being adequately representative of the target RA population in the analyses.

**Table 1:** Response rates general characteristics

		Target population (*n* = 739)	Responders (*n* = 413)	Non-responders (*n* = 324)
Female number (%)		552 (74.7)	306 (74.1)	246 (75.9)
Age mean years (SD)		62.6 (13.6)	63.5 (12.8)	61.5 (14.6)
Social deprivation (IMD LSOA categories)^a^ Number (%)	1 (least deprived)	179 (24.2)	116 (28.1)	63 (19.4)
	2	290 (39.2)	143 (34.6)	147 (45.4)
	3	163 (22.1)	99 (24.0)	64 (19.8)
	4	66 (8.9)	36 (8.7)	30 (9.3)
	5 (most deprived)	41 (5.5)	19 (4.6)	22 (6.8)
Site 2 (%)		385 (52.1)	204 (49.4)	181 (55.8)

^a^IMD scores converted into categories

### Clinical characteristics responders

A wide range of disease duration and current disability was reported (Table 2). The majority of responders were taking medications for their RA (*n* = 394, 95.4%) and one half was taking more than one category of arthritis medication (*n* = 210, 50.8%). Impact of foot problems was substantial with 178 (44.5%) reporting moderate foot related impairment and 105 (26.3%) reporting severe foot related impairment (FIS_IF_). The impact of foot problems in relation to activity levels and participation in valued activates (FIS_AP_) was 110 for moderate impact (27.5%) and 161 (40.3%) for severe. Of the 413 responders 254 (61.5%) were working, of these 156 (61.4%) reported foot problems impacting on their ability to work. The majority (*n* = 312, 75.7%) of responders reported having had a foot examination.

**Table 2:** Clinical characteristics responders

Demographic and clinical characteristics responders (*n* = 413)		
Disease duration median years (IQR)		10 (5 to 20)
Age at diagnosis mean years (SD)		50.3 (14.9)
Arthritis medications number (%)	NSAIDs	128 (31.0)
	DMARDs	339 (82.1)
	Glucocorticoids	122 (29.5)
	Biologics	74 (17.9)
HAQ median score (IQR)^b^		1.5 (0.75 to 2.0)
FIS median score (IQR)^a b^	FIS_IF_	10 (6 to 14)
	FIS_AP_	16 (7 to 23)
Importance median score (IQR)^a^		6 (3 to 8)
Cope median score (IQR)^a^		5 (3 to 7)
Severity median score (IQR)^a^		6 (3 to 8)
Impact of foot problems on ability to work number (%)	Yes	156 (37.8)
	No	98 (23.7)
	Not applicable	159 (38.5)
Time interval of last recalled foot examination number (%)^b^	0–6 months	89 (21.6)
	> 6–12 months	61 (14.8)
	> 12–18 months	34 (8.3)
	> 18 months	97 (23.5)
	Can’t remember	31 (7.5)
	Never had feet examined	100 (24.3)

^a^ *FIS*_*IF*_ Foot Impact Score foot impairment/ footwear restriction subscale, *FIS*_*AP*_ FIS activity limitation/participation restriction subscale; Importance = importance of foot problems; Cope = ability to cope with foot problems; Severity = severity of foot problems

^b^HAQ admissible *n* = 404; FIS admissible *n* = 400; foot examination admissible *n* = 412; IQR = Interquartile range

Almost all respondents reported they had experienced foot problems at some time being diagnosed with RA (Table 3). The majority of responders (*n* = 377, 91.2%) reported the presence of one or more foot problems currently and over half reported (*n* = 215, 52.1%) 5 or more foot problems. The rates of current foot problems in women and men were similar (*n* = 279, 90.0% v *n* = 91, 85.8%).

**Table 3:** Reported foot problems^a^

Category	Foot problem	Current	Ever	Never
Articular features	Pain	263 (63.7)	342 (82.8)	71 (17.2)
	Stiffness	224 (54.2)	277 (67.1)	73 (17.8)
	Swelling	218 (52.8)	286 (69.2)	127 (30.8)
	Any articular feature	305 (73.8)	373 (90.3)	40 (9.7)
Cutaneous lesions	Blisters	28 (6.8)	73 (17.7)	340 (82.3)
	Callus	171 (41.4)	206 (49.9)	297 (50.1)
	Corns	72 (17.4)	109 (26.4)	304 (73.6)
	In-grown toe nails	59 (14.3)	106 (25.7)	307 (74.3)
	Thickened Toe nails	168 (40.7)	190 (46.0)	223 (54.0)
	Ulcers	13 (3.1)	38 (9.2)	375 (90.8)
	Any cutaneous lesions	270 (65.4)	303 (73.4)	110 (26.6)
Structural deformity	Bunions	111 (26.9)	141 (34.1)	272 (65.9)
	Fallen arches	93.(22.5)	121 (29.3)	292 (70.7)
	Misshaped toes	180 (43.6)	199 (48.2)	214 (51.8)
	Any structural deformity	238 (57.6)	265 (64.2)	148 (35.8)
Extra-articular features	Nodules	96 (23.2)	122 (29.5)	291 (70.5)
	Numbness	118 (28.6)	153 (37.0)	260 (63.0)
	Any extra articular feature	176 (42.6)	212 (51.3)	201 (48.7)
Other	Infection	31 (7.5)	76 (18.4)	337 (81.6)
Any foot problems		377 (91.2)	403 (97.6)	10 (2.4)

^a^(*n* = 413 (%))

Of the 413 responders, 287 (69.5%), had accessed foot care (AFC) and 140 (48.8%) had accessed two or more services. The general, clinical and foot related characteristics of the AFC group and the NAFC group are presented in Additional file 1 (online). Overall the AFC group were slightly older and had longer disease duration than the NAFC group (64.9 years v 60.4 years, *p* = 0.01; 12 years v 7 years, *p* < 0.001). Additionally a higher proportion of females had accessed foot care compared to males (F 72.2% v M 61.7% *p* = 0.04). The proportions from more deprived areas (IMD score categories 3, 4 and 5) were similar for both groups (AFC 36.2% v NAFC 39.7% *p* = 0.363).

Although disability was statistically significant in univariate analyses (AFC 1.62 v NAFC 1.12 *p* = 0.005), HAQ scores included lower limb disability and these were therefore omitted from multivariate analyses. As an assumption of logistic regression analyses requires independent variables are independent of the dependent variable of interest [32]. Logistic regression analyses revealed increased odds (OR) of AFC in those with longer disease duration (OR = 1.04, 95% CI 1.02–1.07) and in those who were older (OR = 1.02, 95% CI 1.01–1.04). Although these results demonstrate disease duration and age to be independent factors of access to foot care the effect for both was mild. The strongest predictor of AFC was female gender, with an odds ratio of 1.72 (95% CI 1.06–2.88). Social deprivation did not make a statistically significant contribution to the model (OR 0.87, 95% CI 0.70–1.09). Table 4 presents the results of univariate and logistic regression analyses.

**Table 4:** Independent variables determinants AFC (n = 413)

Predictive variable	Univariate analyses^a^	Multivariate analyses^b^	Exp (B) Adjusted odds ratio (CI 95%)^c^	*p*
Hospital site	Proportions similar AFC versus NAFC according to hospital site	Excluded from model		
Gender	*p* = 0.04	Included in model	1.72 (1.06–2.88)	0.03
Age	*p* = 0.01	Included in model	1.02 (1.00–1.04)	0.02
Social deprivation	*p* = 0.363	Excluded from model		
Disease duration	*p*= > 0.001	Included in model	1.04 (1.02–1.07)	< 0.01
Arthritis medications	Proportions similar AFC versus NAFC according to arthritis medications	Excluded from model		
Disability (HAQ)	*p* = 0.005	Exclude from model, as 1 section captures lower limb disability		

^a^Univariate analyses of demographic and clinical variables to identify factors associated as predictors of AFC

^b^Variables with p = < 0.2 in univariate analyses were entered into a series of logistic regression models to identify independent predictors of AFC

^c^CI 95% Confidence Interval

The AFC group reported a wide range of foot care received (Table 5). Device prescriptions were the most frequent care category reported. Of the 72 patients who had undergone foot surgery, 54 (75.0%) reported their foot problems had improved after surgical intervention.

**Table 5:** Foot care interventions received

Foot care category	Foot care interventions	AFC (*n* = 287)^a^ Number (%)
Devices	Hospital shoes	73 (25.4)
	Insoles	190 (66.2)
	Padding	58 (20.2)
	Toe protectors	11 (3.8)
	Any devices	222 (76.4)
Non-pharmacological	Advice	69 (24.0)
	Foot exercises	42 (14.6)
	Any non-pharmacological	93 (32.4)
Pharmacological	Antibiotics for infection	24 (8.4)
	Creams for infection	22 (7.7)
	Steroid injection	40 (13.9)
	Any pharmacological	74 (25.8)
Treatment cutaneous lesion	Nail care	53 (18.5)
	Treatment for corn/callus	62 (21.6)
	Wound care	19 (6.7)
	Any treatment cutaneous lesions	99 (34.5)
Other	Foot surgery	72 (25.1)

^a^Missing data 9 (3.1%) AFC group

Of the AFC group, 204 (71.1%) had accessed podiatry (NHS and/or independent sector), 192 (66.7%) orthotics and 92 (32.1%) orthopaedics and 140 (48.8%) had accessed two or more services (Table 6). Over half of the podiatry group had accessed independent sector podiatry care (with or without NHS care) (*n* = 107, 52.5%). No information was available on exclusive use of independent sector care. Of the patients who had accessed podiatry, nearly half (*n* = 95, 46.5%) had self-referred including 39 (19.1%) who had accessed independent sector foot care (i.e. outside the NHS). There were differences in access routes for each service according to gender. Women were more likely than men to access podiatry (NHS and/or independent sector) care through self-referral (29.7% v 20.0%) and from hospital clinicians (26.6% v 17.4%) whereas men were more likely to be referred by their GP (41.3% v 22.2%). The proportions of patients self-referring for independent sector podiatry care was similar for both genders. In contrast the proportion of women referred to orthopaedics by hospital based clinicians was higher than GP initiated referrals (F 71.4% v F 20.7%). Access routes to orthotics were broadly similar for gender.

**Table 6:** Gender and routes of access to foot care services^a^

Referral route		Podiatry (*n* = 204) (Number (%)		Orthotics (*n* = 192) Number (%)		Orthopaedics (*n* = 92)Number (%)	
		Female *n* = 158 (77.5)	Male *n* = 46 (22.5)	Female *n* = 151 (78.6)	Male *n* = 41 (23.4)	Female *n* = 77 (83.7)	Male *n* = 15 (16.3)
Self	Any podiatry	47 (29.7)	9 (20.0)	3 (2.0)	0 (0)	1 (1.3)	0 (0)
	Independent	30 (19.0)	9 (20.0)	2 (1.3)	0 (0)	2 (2.6)	1 (6.7)
GP		35 (22.2)	19 (41.3)	19 (12.6)	6(14.6)	16 (20.7)	9 (60.7)
Hospital		42 (26.6)	8 (17.4)	126 (83.4)	33 (80.2)	55 (71.4)	5 (33.3)
Community nurse		2 (1.3)	0 (0)	0 (0)	0 (0)	1 (1.3)	0 (0)
Other		2 (1.3)	1 (2.2)	1 (0.7)	2 (1.0)	2 (2.6)	0 (0)
Missing data		0 (0)	0 (0)	1 (0.7)	0 (0)	0 (0)	0 (0)

^a^ ≥ 2 services accessed

## Discussion

This study achieved an overall response rate of 56%, similar to other postal surveys of patients with RA conducted in the UK [4, 33]. Responders and non-responders were similar in relation to hospital site, gender, age; and social deprivation. Additionally the general and RA characteristics of responders were similar to other postal surveys of patients with RA conducted in the UK [7, 33–35]. Thus the responders to this survey are likely to represent the target population, and the target population may reflect the general diagnosed RA population.

These results show that patients with RA in this study experience a wide range of foot problems and the great majority currently have one or more foot problems. The prevalence of foot pain, numbness and ulceration reported in this study were broadly similar to but slightly lower than earlier surveys based on selected populations [4, 7]. However, this study provides the broadest description of foot problems reported in a random sample of a defined population of patients with RA to date.

Foot impact scores were slightly lower than previous studies [5, 36, 37]. However, these data represent the overall impact of foot problems of all patients with RA in a defined geographical area as opposed to selected samples such as patients attending a specific clinic. The FIS scores in this study show the impact of foot problems to be considerable in a wide population sample of patients with RA. However, the findings of an earlier qualitative study indicated not all issues of the impact of foot problems (e.g. work related disability) are captured in the FIS [28]. Work related disability in patients with RA has been widely described [38–40] and this study provides new insights identifying the specific and substantial impact of foot problems on work related disability.

An annual review of patients’ feet and access to foot care services is recommended in national guidelines [12]. However, surveys of rheumatology departments have shown the provision of dedicated foot care services for patients with inflammatory arthritis is variable [15–17]. Additionally, the conduct of foot examinations in clinical practice can also be variable as the foot is omitted from standard measures of disease activity such as the Disease Activity Score [41] and consequently foot problems may be ignored in clinical consultations [2, 11]. The majority of responders in this study recalled having undergone a foot examination, although only a third reported to having had a foot examination in the preceding 12 months. The accuracy of patients recalling the time when foot examinations were conducted can be questioned. Nonetheless this study suggests patients perceive the time interval of when foot examinations were conducted to be variable and potentially not complying with national guidelines [12].

Based a review of the literature and local service evaluation, it was estimated that many patients within the study geographical area would not have accessed foot care. However, the proportion of patients who had accessed foot care was higher than anticipated. It is possible repeated interactions with clinicians may overcome barriers to utilisation of health care services and thus patients with RA may be more likely to access additional services including foot care. Indeed patients with RA being regular users of health care provided by a wide range of clinicians has been reported [42]. However, access to and utilisation of health care services in general terms is complex. A number of predisposing factors such as general characteristics (age, gender, social deprivation), clinical characteristics (type of health condition acute or long term, additional morbidity) experience and satisfaction of care received are reported to influence individuals in their decisions to access health care or not [43]. Furthermore, patients’ reasons for accessing and utilising foot care services are complex [22]. In spite of the higher than expected access rates, many patients from both the AFC group and NAFC group reported current foot problems. The proportion of patients who had accessed podiatry was similar to an earlier postal survey of patients with RA [4]. However, in a longitudinal cohort study of patients with early RA, only 30% of study participants reported to have accessed podiatry and 4% orthopaedics [20]. This may be accounted for by the differences between this random RA-population sample compared to an early arthritis cohort.

In multivariate analyses age and disease duration were independently associated with having accessed foot care, but not strongly. Foot problems are reported to affect one in four older people [44]. It therefore is possible to postulate patients with RA who are older and have longer disease duration are more likely to develop foot problems and therefore seek foot care. However, severity and clinical progression of RA varies from patient to patient. Thus the mild influence of age and disease duration as possible determinants of accessing foot care would seem reasonable. Female gender was the strongest independent predictor of AFC. This is not an unexpected result as it has been reported foot problems may be more prevalent in women [45, 46], and utilisation of health care has been widely reported to be higher in women [47–49]. This study has identified broad characteristics of which patients are likely to access foot care in in Bristol, UK. For example women were more likely to self-refer and men more likely to be advised by clinicians to seek treatment. Knowing that female patients are more likely to access foot care does not necessarily reflect greater clinical need.

In the UK, podiatry services are provided within both the NHS and independent health care sectors. Of the responders who had accessed podiatry services one half reported to have accessed independent sector foot care. A recent UK based survey of 217 patients with RA who had accessed podiatry care showed that the majority (76%) had used independent sector care [21]. It could be inferred that patients consider their foot problems to be an important health care need that they are willing to self-fund. Conversely, patients may elect to self-fund their foot care due to lack of NHS provided foot care, barriers to accessing care and/or experience of care received [22].

Of the AFC group, a range of care interventions was reported. Foot care “devices” was the most common care category reported suggesting clinicians consider them beneficial. However, despite having accessed foot care, the majority of patients reported current foot problems and substantial impact. These data therefore raise questions regarding effectiveness and/or continuity of foot care received. However, data in relation to the clinical significance of foot problems at the time foot care was accessed is not available. Firm conclusions regarding the effectiveness of foot care received cannot be drawn directly from these data, as that would require contemporaneous clinical examination of the feet.

The number of podiatrists within the independent sector specialising in inflammatory arthritis within the geographical area of this study is unknown. However, non-specialists’ knowledge of national guidelines for the management of foot problems in rheumatic diseases shows wide variation [50]. In order for patients to receive timely and effective care non-specialist clinicians need to have the clinical skills to identify and refer patients who require specialist review. The role of specialist podiatrists as members of hospital based multidisciplinary teams’ managing diabetic foot disease is well established in the UK [51]. In contrast, the number of podiatrists as members of rheumatology multidisciplinary teams in the UK is variable [15]. Provision of foot care services for patients with RA could reflect the diabetes model. Indeed close collaboration between clinicians for access and management of foot problems in RA has long been advocated [52].

Overall, this study has provided data in relation to: (i) a description of the general and RA characteristics of a large random sample of all RA patients residing within a defined geographical area; (ii) estimated the reported prevalence and impact of current foot problems; (iii) identified the proportion of patients who have accessed foot care services; (iv) provided a description of foot care patients received. These data are from a community based random sample of all patients with RA in a defined geographical area. How and whether or not patients in the general RA population access foot care may be variable and potentially influenced by different patterns of foot care service provision. How these data reflect the situation in other geographical areas could be the focus of further investigation.

### Strengths and limitations

Is a strength of this study that the patient population was a random sample of all patients within a defined geographical area, the sample size was large and responders were an accurate representation of the target population of adult patients with RA. Additionally, almost all the returned questionnaires were admissible (minimal missing data), indicating patients found the survey items easy to understand and complete. There are some limitations to this study. While the prevalence of one or more current foot problems in patients with RA by self-report was very high, it would be useful to know to what extent patient self-report is accurate. The clinical categorisation of foot problems and the proportion of patients who might benefit from immediate foot care could be identified by direct clinical examination of the feet. Data relating to the prevalence of foot problems in non-responders is not known and there is potential for non-responder bias. However, in this study the general characteristics of responders and non-responders that might cause such bias, such as the proportions of responders from areas of deprivation, were very similar.

## Conclusion

Self-reported foot problems were present in 91% of a random sample all of adult patients with RA in Bristol and they substantially impacted on patients’ lives. Additionally foot problems were important issues for patients. While overall access to foot care was higher than anticipated, the extent of current problems suggests that the provision of effective, timely and targeted care a pressing need. Clinicians need to have the clinical expertise in foot assessments and knowledgeable of the clinical management of foot problems. Additionally foot care needs to be co-ordinated and tailored to individual patient’s needs in order to improve outcomes for patients.

## Additional file

Additional file 1: Characteristics AFC versus NAFC. (DOCX 13 kb)

## Abbreviations

CCG: Clinical commissioning group
FIS: Foot impact scale
HAQ: Health assessment questionnaire
IMD: Index of multiple deprivation
NHS: National health service
RA: Rheumatoid arthritis
UK: United Kingdom
