# Supplementary material for: Prevalence, impact and care of foot problems in people with rheumatoid arthritis: results from a United Kingdom based cross-sectional survey
Source: J Foot Ankle Res. 2017 Oct 27;10:46. doi: 10.1186/s13047-017-0229-y (PMC5658923; doi:10.1186/s13047-017-0229-y)
Supplement: Additional file 1: — Characteristics AFC versus NAFC. (DOCX 13 kb) [file 13047_2017_229_MOESM1_ESM.docx]

**Additional File 1 Characteristics AFC versus NAFC**

|  | | **AFC (n=287)** | **NAFC (n=126)** | **P** |
| --- | --- | --- | --- | --- |
| Gender | Female number (%) | 221 (72.2) | 85 (38.5) | p=0.04 |
| Age mean years (SD) |  | 64.9 (12.5) | 60.4 (13.0) | p=0.01 |
| Social deprivation  (IMD LSOA categories)  Number (%) | 1 (least deprived) | 89 (31.0) | 27 (21.4) | p=0.363 |
|  | 2 | 94 (32.8) | 49 (38.9) |  |
|  | 3 | 67 (23.3) | 32 (25.4) |  |
|  | 4 and 5 (most deprived) | 37 (12.9) | 18 (14.3) |  |
| Hospital site | UHB number (%) | 138 (67.6) | 66 (32.4) |  |
| Disease duration median years (IQR) |  | 12 (6 to 21) | 7 (3 to 12) | p<0.001 |
| Arthritis medications number (%)* | NSAIDs | 93 (32.4) | 35 (27.8) |  |
|  | DMARDs | 230 (80.1) | 109 (86.5) |  |
|  | Glucocorticoids | 79 (27.5) | 43 (34.1) |  |
|  | Biologics | 51 (17.7) | 23 (18.3) |  |
| HAQ median score (IQR) † |  | 1.65  (0.8125 to 2.0) | 1.125  (0.5 to 1.875) |  |
| FIS median score (IQR) † | FIS_IF_ | 11 (7 to 14) | 8 (4 to 12) |  |
|  | FIS_AP_ | 17 (7 to 23) | 13.5 (7 to 23) |  |
| Importance median score (IQR) |  | 7 ( 4 to 9) | 5 (2 to 8) |  |
| Cope median score (IQR) |  | 5 (3 to 7) | 4 (1 to 6) |  |
| Severity median score (IQR) |  | 6 (4 to 8) | 5 (2 to 7) |  |
| Impact of foot problems on ability to work number (%) | Yes | 103 (62.8) | 53 (58.9) |  |
| Current foot problem categories (%) † | Articular features | 229 (76.7) | 85 (67.5) |  |
|  | Cutaneous lesions | 206 (71.8) | 64 (50.8) |  |
|  | Structural deformity | 184 (64.1) | 54 (42.8) |  |
|  | Extra articular features | 134 (46.7) | 42 (33.3) |  |
|  | Other (infection) | 25 (8.7) | 6 (4.8) |  |
|  | Any problems | **269 (93.7)** | **101 (80.2)** |  |

*FISIF = Foot Impact Score foot impairment/ footwear restriction subscale; FISAP = FIS activity limitation/participation restriction subscale; Importance = importance of foot problems; Cope = ability to cope with foot problems; Severity = severity of foot problems
